# Room-temperature synthesis of nanometric and luminescent silver-MOFs

**DOI:** 10.3389/fchem.2022.1065622

**Published:** 2023-01-06

**Authors:** Vanessa Celis-Arias, Ismael A. Garduño-Wilchis, Gilberto Alarcón, Fernando González Chávez, Efrain Garrido Guerrero, Hiram I. Beltrán, Sandra Loera-Serna

**Affiliations:** ^1^ Departamento de Ciencias Básicas, Universidad Autónoma Metropolitana Azcapotzalco, Ciudad de México, Mexico; ^2^ Cátedras CONACyT, Instituto Politécnico Nacional, Centro de Investigación en Ciencia Aplicada y Tecnología Avanzada, Ciudad de México, Mexico; ^3^ Instituto Politécnico Nacional, Centro de Investigación en Ciencia Aplicada y Tecnología Avanzada, Ciudad de México, Mexico; ^4^ Universidad Politécnica Del Estado de Nayarit, Tepic, Nayarit, Mexico; ^5^ Departamento de Genética y Biología Molecular, Centro de Investigación y de Estudios Avanzados del Instituto Politécnico Nacional, Ciudad de México, Mexico

**Keywords:** silver MOFs, coordination polymer, luminescence, stability, persistence

## Abstract

Three silver-MOFs were prepared using an optimized, room-temperature methodology starting from AgNO₃ and dicarboxylate ligands in water/ethanol yielding **Ag**_**2**_ **BDC**, **Ag**_**2**_ **NDC** (**UAM-1**), and **Ag**_**2**_ **TDC** (**UAM-2**) at 38%–48% (BDC, benzenedicarboxylate; NDC, 1,8-naphthalene-dicarboxylate; TDC, p-terphenyl-4,4″-dicarboxylate). They were characterized by PXRD/FT-IR/TGA/photoluminescence spectroscopy, and the former two by SEM. These materials started decomposing at 330°C, while showing stability. The crystal structure of **UAM-1** was determined by PXRD, DFT calculations, and Rietveld refinement. In general, the structure was 3D, with the largest Ag-O bond interlinking 2D layers. The FT-IR spectra revealed 1450 and 1680 bands (cm^−1^) of asymmetrically stretching aniso-/iso-bidentate -COO in coordination with 2/3-Ag atoms, accompanied by Ag-O bands at 780–740 cm^−1^, all demonstrating the network formation. XRD and SEM showed nanometric-scale crystals in **Ag₂BDC**, and **UAM-1** developed micrometric single-stranded/agglomerated fibrillar particles of varying nanometric widths. Luminescence spectroscopy showed emission by **Ag₂BDC**, which was attributed to ligand-to-metal or ligand-to-metal–metal transitions, suggesting energy transfer due to the short distance between adjacent BDC molecules. **UAM-1** and **UAM-2** did not show luminescence emission attributable to ligand-to-metal transition; rather, they presented only UV emission. The stabilities of **Ag₂BDC** and **UAM-1** were evaluated in PBS/DMEM/DMEM+FBS media by XRD, which showed that they lost their crystallinity, resulting in AgCl due to soft–soft (Pearson’s principle) affinity.

## 1 Introduction

Silver nanoparticles (AgNPs) have been extensively used for numerous applications (Eckhardt et al., 2013; Alarcon et al., 2015). In particular, the biocidal effect of silver has been studied for several years (Antelman 1991; Morones et al., 2005; Fromm 2011; Eckhardt et al., 2013; Zhang et al., 2022). Silver has antibacterial properties that improve when it is structured at the micro or nanometric scale; moreover, these small particles are more efficient than raw or bulk metallic silver. Indeed, silver nanostructures show size-dependent antibacterial capacities, with trends for increased antibacterial effects with decreasing silver nanoparticle size (Pal et al., 2007; Belser et al., 2009; Sayen 2014; Cao 2017; Zhang et al., 2022). Based on these findings, commercial products have been developed, which use AgNP to eliminate bacteria in food (Cao 2017); inhibit surface bacterial colonization through immobilized AgNP (Wirth et al., 2016); and anti-bacterial, anti-biofilm, and wound-healing, in addition to bioimaging technologies for AgNP coated with pectin (Pallavicini et al., 2017). Another application for silver-based materials is the use of MOF-type structures for biocidal agents (Wyszogrodzka et al., 2016) due to the slow release of ions or fragments from the network (Chiericatti et al., 2012) or the biocidal properties of the material (Zhuang et al., 2012). Therefore, the persistence of these materials has been studied for these possible applications. The first silver coordination polymers were established in the 1990s (Sagatys et al., 1992; Smith et al., 1995; Smith et al., 1996). Subsequently, nano-sized AgMOFs and related structures were developed for different applications (Arenas-Vivo et al., 2019; Li et al., 2020; Arenas-Vivo et al., 2021).

Among these, the field of luminescent MOFs (LMOFs) has attracted increased interest in recent years through the development of numerous new examples of LMOF materials, with chemical structures and physical properties adapted to different applications. The development of these types of nanomaterials is at the forefront of nanoscience and nanotechnology, the latter due to their potential applications with luminescent properties including light-emitting diodes (LEDs); photonics sensors; visible light communications; sensing devices for pollutants or volatile organic compounds (VOCs); or simple as pH, pressure, or temperature sensors (Brites et al., 2018; Zhang et al., 2018; González Chávez et al., 2020; Gutiérrez et al., 2020; Garduño-Wilches et al., 2021). One important characteristic among these compounds is been their response to external stimuli, which has attracted research interest and development (Zhang et al., 2018). Several Ag-coordinated polymers have shown luminescence capabilities, some of them reported years ago (Ding et al., 2005); however, their emissive behavior is not well understood, with some cases showing ligand-to-metal energy transfer (through bonds) and others showing antenna effects (through space). Moreover, their emissive contributions “turn on” or vary due to particular structural arrangements between ligands, for e.g., carboxylate coordination modes, toward the Ag centers, and the number of Ag atoms in the coordinating net, but also due to the number and positions of carboxylate moieties in the linker, with emissive examples of two, three, four, or even six carboxylate moieties in the ligand (Sun et al., 2014; Xia et al., 2018; Gutiérrez et al., 2020; Yu et al., 2020; Chen et al., 2021; Mackenzie et al., 2021).

The present study synthesized three silver MOFs through a room temperature modification of the methodology proposed by Sun et al. (Sun et al., 2004). Therein, the authors obtained, among other materials, the **Ag**_**2**_ **BDC** framework (benzene-1,4-dicarboxylic acid, BDC) that showed short Ag–Ag contacts, which crystallized in a monoclinic system with P 2_1_/c space group in a 1-week synthetic procedure. The present study synthesized the same **Ag**_**2**_ **BDC** material with the same structure in a shortened room temperature procedure lasting just 24 h, using EtOH and water as a solvent mixture and with a slightly higher yield. In addition, the size of the crystals obtained by the optimized synthetic methodology achieved a nanometric-sized **Ag**_**2**_ **BDC** version. In a similar fashion to **Ag**_**2**_ **BDC** other two dicarboxylate ligands were employed; NDC (naphthalene-2,6-dicarboxylic acid) and TDC ([1,1′:4′,1″-terphenyl]-4,4″-dicarboxylic acid), which yielded two new AgMOFs: **Ag**_**2**_ **NDC** (**UAM-1**) and **Ag**_**2**_ **TDC** (**UAM-2**), also with moderate yields employing the new modified synthetic procedure. These three AgMOFs were physicochemical and spectroscopically characterized by scanning electron microscopy (SEM), powder X-ray diffraction (PXRD), Fourier transform infrared spectroscopy (FT-IR), thermogravimetric analysis (ATG), N_2_ adsorption, and luminescence spectroscopy. To our knowledge, this is the first time that these three AgMOFs have been synthesized on the nanometric scale, and the first time they were evaluated for their persistence in aqueous media and culture media. The MOFs were (non)persistent depending on the treatment conditions, without the “common” belief of Ag^+1^ release; therefore, they acted as the main species responsible for biological activities. These AgMOF-based materials may show potential in many biomedical applications due to the evidence presented in the present study.

## 2 Experimental section

**Materials:** All the chemicals were used as received. Benzene 1,4-dicarboxylic acid (**BDC**, MW: 166.13 g mol^−1^, 98% grade), 2,6-naphthalene-dicarboxylic acid (NDC, MW: 216.19 g mol^−1^, 99% grade), p-terphenyl-4,4″-dicarboxylic acid (TDC, MW: 318.32 g mol^−1^, 90% grade), sodium hydroxide (NaOH, MW: 166.13 g mol^−1^, 98% grade), and silver nitrate (AgNO_3_, MW: 169.87 g mol^−1^, 99% grade) were purchased from Sigma-Aldrich. Anhydrous ethanol (EtOH, 99% of purity, Aldrich) and deionized water were used as solvents.

Three AgMOFs were prepared: **Ag**_**2**_ **BDC**, **Ag**_**2**_ **NDC** (**UAM-1**), and **Ag**_**2**_ **TDC** (**UAM-2**). For general purposes, only the **Ag**_**2**_ **BDC** synthesis procedure is described. The other two materials were prepared accordingly. Additionally, an experimental design including a synthesis methodology that varied factors from stirring at room temperature (STR), ultrasound (US)-assisted, or solvothermal (ST) methods, as well as Ag: BDC molar ratios of 1:1, 1:2, and 2:1 were surveyed for the **Ag**_**2**_ **BDC** case to optimize the reaction conditions to yield the desired material, which was ascertained by XRD (Supplementary Figure S1). The conditions with 2:1 stoichiometries yielding the desired material under STR or US were used. To provide a simple synthetic strategy, only the STR method is described.

**Ag**_**2**_ **BDC synthesis:** The synthesis was performed as reported by Sun et al. (Sun et al., 2004), with important modifications. First, 3 mmol of BDC and 7.5 mmol of NaOH were dissolved in 80 ml of deionized water in a flask/vial. Then, a solution containing 3 mmol of AgNO_3_ and 20 ml of anhydrous ethanol was added dropwise, and the reaction mixture was stirred at room temperature (r.t.) overnight. The **Ag**_**2**_ **BDC** product was isolated as a nanocrystalline material by centrifugation (4032 g RCF, 10 min) that was dried at 323 K for 12 h. The yield was determined as a dry basis corrected through thermogravimetric analysis (TGA).

**Ag**_**2**_ **BDC:** Brown solid, yield 43%. FT-IR (ν, cm^−1^): 3094, 3070 (C_Ar_-H), 1529 (COOAg_2_), 1492 (COOAg_3_), 1358 (C_COO_-OAg), 1148 (C-C-H), 1090 (C-C-H), 1013 (C-C-H), 887 (C-C-H), 818 (C-C-H), 740 (Ag-O), 530, 448 (O-Ag-O). PXRD (2θ, °, (intensity)_[h k l]_): 13.2 (1053)_[1 0 0]_, 16.6 (950)_[1 1 0]_, 18.8 (1007)_[1 1 -1]_, 25.1 (901)_[0 2 1]_, 25.6 (920)_[1 1 1],_ 28.3 (935)_[2 1 0],_ 30.9 (1282)_[1 2 1]_, 32.2 (990)_[2 2 -1]_, 34.2 (952) _[1 3 -1]_, 40.6 (1001)_[2 3 0],_ 42.6 (944) _[1 4 0]._ BET (m^2^·g^−1^): 4.94. TGA (%w, T_interval_ [°C]): 0%, 25–330; 42%, 330–450; 0%, 450–800. Calculated elemental analysis for C₈H₄O₄Ag₂ %C, 25.30; %H, 1.06; %O, 16.85; %Ag, 56.79. The elemental analysis was performed by TGA (%C+%H+%O) = 42, (%Ag) = 58.

**Ag**_**2**_ **NDC (UAM-1):** Brown solid, yield 48%. FT-IR (ν, cm^−1^): 3063, 3032 (C_Ar_-H), 1595 (COOAg), 1523 (COOAg_2_), 1487 (COOAg_3_), 1389, 1356 (C_COO_-OAg), 1195 (C-C-H), 1138 (C-C-H), 1101 (C-C-H), 916 (C-C-H), 842 (C-C-H), 789 (C-C-H), 769 (Ag-O), 653 (C-C-H), 632 (C-C-H), 548, 473 (O-Ag-O), 453 (O-Ag-O). PXRD (2θ, °, (intensity)): 10.64 (23280)_[1 0 0]_, 17.53 (4698)_[1 1 0]_, 18.31 (5119)_[0 0 1]_, 19.77 (2770)_[1 -1 0]_, 20.50 (3050)_[0 1 -1]_, 20.86 (3752) _[1 0 -1]_, 21.39 (4362)_[2 0 0],_ 21.60 (3877)_[1 0 1],_ 21.91 (2642)_[1 1 -1],_ 24.40 (4695)_[1 -1 1]_, 24.71 (2851)_[2 1 0]_, 27.04 (2989)_[0 1 1]_, 27.79 (8579)_[2 0 -1]_, 27.83 (7857)_[2 1 -1]_, 28.66 (3988)_[1 1 1]_, 28.71 (3821)_[2 0 1]_, 31.03 (3738)_[0 2 0]_, 31.59 (5222)_[0 2 -1]_, 31.62 (5138)_[2 -1 1]_, 31.66 (5104)_[2 -1 1]_, 31.75 (5125)_[1 2 -1]_, 33.91 (5221)_[2 1 1]_, 34.93 (3296)_[1 -2 1],_ 35.52 (2335)_[2 -1 -1]_, 35.62 (2669)_[2 2 -1]_, 36.17 (2395)_[3 1 -1]_, 36.21 (2463)_[0 1 -2]_, 36.63 (3004)_[3 0 -1]_, 36.82 (3104)_[1 1 -2]_, 38.78 (2038)_[1 -1 2]_, 40.46 (2188)_[2 1 -2]_, 41.11 (2171)_[2 -2 1]_, 41.57 (3679)_[3 1 1]_, 41.65 (3579)_[0 2 -2][1 2 -2]_, 44.39 (2579)_[0 1 2]_, 45.55 (2121)_[1 1 2]_, 45.69 (2384)_[2 2 -2]_, 47.16 (2138)_[1 3 0]_, 49.60 (1963)_[2 3 0]_. BET (m^2^·g^−1^): 11.51. TGA (%w, T_interval_ [°C]): 0%, 25–310; 47.62%, 350–400; .9%, 400–800. Calculated elemental analysis for C₁₂H₆O₄Ag₂ %C, 33.53; %H, 1.41; %O, 14.89; %Ag, 50.18. Determined elemental analysis by TGA (%C+%H+%O) = 47.62, (%Ag) = 52.38. During the synthesis of this material, slight ultrasonication periods of 10 s with a rest time of 2 min, five times were applied to the reaction sine to achieve better mixing of the reagents. The X-ray structure of this material was obtained from the PXRD diffraction file, optimization of the DFT plane wave periodic conditions, and Rietveld refinement, as described later.

**Ag**_**2**_ **TDC (UAM-1):** Brown solid, yield 38%. FT-IR (ν, cm^−1^): 3500-2600 (O-H, H_2_O, EtOH from synthesis); 3028 (C_Ar_-H), 1639, 1598, 1587 (COOAg), 1542, 1529 (COOAg_2_), 1483 (COOAg_3_), 1383, 1281 (C_COO_-OAg), 1191 (C-C-H), 1148 (C-C-H), 1101 (C-C-H), 932 (C-C-H), 867, 834 (C-C-H), 767 (Ag-O), 756 (Ag-O), 687 (C-C-H), 636 (C-C-H), 538, 463 (O-Ag-O). PXRD (2θ, °, (intensity)): 19.55 (4384), 23.51 (3321), 24.23 (3748), 26.91 (3100), 27.84 (7642), 28.68 (3658), 32.26 (12515), 33.03 (15834), 34.19 (4107), 38.30 (6201), 42.37 (2709), 46.28 (5996), 54.92 (3201), 55.38 (3130), 57.52 (2564), 65.85 (2179), 67.47 (1894), 74.59 (1427), 76.73 (1933), 85.73 (1431). BET (m^2^·g^−1^): 6.97. TGA (%w, T_interval_ [°C]): 10.88%, 25–70; 6.92%, 70–130; 18.22%, 130–350; 18.60% 350–470; 26.23% 470–800. Calculated elemental analysis for C₂₀H₁₂O₄Ag₂ %C, 45.15; %H, 2.27; %O, 12.03; %Ag, 40.55. The elemental analysis was performed by TGA (%C+%H+%O) = 76.7, (%Ag) = 23.3.

**PXRD:** All AgMOFs samples were characterized by powder X-ray diffraction (PXRD) analysis. A powder X-ray diffractometer (D8 Advance, Bruker) coupled to a Cu anode X-ray tube with LYNXEYE detector was used to identify the crystalline structure of the materials. Cu *Kα* radiation (λ = 1.5406 Å) was selected, with a diffracted beam monochromator, a step size of .01″, a time per step of .9 s, and 2ϴ scans between 4° and 60°.

**FT-IR:** The Fourier transform infrared (FT-IR) spectra (4000–650 cm^−1^) of the three silver materials were obtained at a resolution of 2 cm^−1^ at r.t. on a Bruker Tensor 27 spectrometer fitted with a DTGS detector. The FTIR spectra were recorded for the raw samples using the attenuated total reflectance (ATR) technique.

**TGA:** The TGA of these three AgMOFs was performed under an N_2_ atmosphere at a rate of 5°C·min^−1^ with on TGA model Q500 (TA Instruments, USA). The samples were heated from room temperature to 800°C at a rate of 10°C·min^−1^.

**SEM:** The scanning electron microscopy (SEM) images of **Ag**_**2**_ **BDC** and **UAM-1** were obtained on a Supra 55VP microscope (Carl Zeiss, Oberkochen, Germany) and measured without covering by direct atmospheric determination.

**Textural properties**: The adsorption measurements were conducted using a BELSORP-max (BELL Japan Inc.) system at −196°C. The samples were degassed under dynamic conditions (extra-dry airflow) over 24 h at 100°C before N_2_ adsorption measurements. The BET-specific surface areas were calculated from the N_2_ adsorption isotherms.

**Stability test:** The structural stabilities **Ag**_**2**_ **BDC** and **UAM-1** were determined by setting a 4000 mg L^−1^ concentration in deionized water (milli-Q), PBS (phosphate-buffered saline), DMEM (Dulbecco′s modified Eagle′s medium), and DMEM+FBS (fetal bovine serum) (Ruyra et al., 2015). The samples were maintained with slow and constant stirring for 24 h at 4, 37°C, and r.t. Later, the solids were isolated by centrifugation at 4032 g RCF for 10 min. Finally, the remnant solids were dried at 50°C for 18 h and characterized by PXRD.

**Luminescent properties:** UV-vis absorbance and all photoluminescence studies were performed on an FS5 spectrophotometer (Edinburgh Instruments). Different modules were used, including an SC-30 module with an integrating sphere to acquire absorptance spectra and an SC-15 module with indirect excitation optics for the acquisition of excitation and emission spectra.

**DFT X-ray structure calculations:** Theoretical DFT calculations of the **UAM-1** structure were carried out in Quantum ESPRESSO (v.6.4.1) (QE), an open-source suite for the quantum simulation of materials (Giannozzi et al., 2009; Giannozzi et al., 2017), using a plane wave self-consistent field (PW_SCF_) program employing projector-augmented wave (PAW) pseudopotentials for electron-core shell (Kresse and Joubert, 1999) and exchange-correlation scalar relativistic functional employing Perdew–Burke–Ernzerhof (PBE) (Perdew et al., 1996) (pbe-kjpaw_psl.1.0.0. UPF, as referenced on the QE site: https://www.quantum-espresso.org/pseudopotentials/original-qe-pp-library/o). Due to the particular Ag···Ag and Ag···O bonding schemes in these types of materials, two van der Waals/dispersion approaches were analyzed, the Grimme DFT-D3 (Grimme et al., 2010), and the Grimme D2 (Grimme, 2006), both on which introduced semiempirical corrections to acquaint long-range vdW interactions and dispersion schemes. To assess the effect of the set of parameters used to achieve convergence, energy cutoffs of rho at 450, 650, 850, 1050, 1250, and 1500 Ry, and the corresponding energy cutoffs for wavefunction at 45, 65, 85, 105, 125 and 150 Ry, were employed. A force convergence threshold of 1·10^−3^, mixing beta of .4, and automatic k points 1 2 2 0 0 0 were also used for all calculations. The theoretical calculations of **UAM-1** were obtained using the fixed cell relaxation procedure starting from the indexed cell obtained by the EXPO14 Rietveld Refinement program suite (Altomare et al., 2018). The structural coordinates of half of the molecular entity were modified from the single-crystal X-ray structure experimentally obtained for **Ag**_**2**_ **BDC** through the replacement of half **BDC** with half **NDC** ligands. All calculations were carried out in the QE parallel version (MPI and OpenMP), running on 128 processor cores in the Cluster Yoltla hosted at Laboratorio de Supercómputo y Visualización en Paralelo (LSVP) UAM-Iztapalapa (http://www.lancad.mx/?page_id=96).

**Rietveld X-ray powder pattern refinement:** Rietveld refinement was performed in the EXPO14 Rietveld Refinement program suite (Altomare et al., 2018) using the complete experimental X-ray diffractogram of the **UAM-1** (2θ range of 6°–70°) powder sample. The cell and peak shape parameters (Pearson VII function) were finally refined with the structure obtained by DFT calculations in QE. The P 1 space group was set for the DFT calculations and the **UAM-1** geometrically optimized structure was loaded but with the P -1 (inversion center space group) for the automatic refinement of the experimental profile, including the non-structural parameters with the Le Bail method (Le Bail et al., 1988). The three weighting schemes in EXPO14 (w = 1; w = 1·counts^−1^; w = 1·counts^−2^) were applied to ascertain the minimal statistical “r” for the refinement; in all cases, the “w = 1·counts^−1^” was the best weighting scheme for this structure.

## 3 Results and discussion

This study synthesized three silver MOFs—**Ag**_**2**_ **BDC** (43% yield), **UAM-1** (48% yield), and **UAM-2** (38% yield)—using a modification of the procedure described by Sun et al, which provided moderate yields (Sun et al., 2004). The present study synthesized these materials using a simpler procedure performed at room temperature, in 1/7 of the original reaction time, and with nearly the same yield (in reference to the **Ag**_**2**_ **BDC** material) compared to the reported procedure, which used a hot mixture of water and methanol as reaction media and required 1 week (Sun et al., 2004). The main variation was the conjoint use of an EtOH: water solvent mixture as the reaction media at room temperature. The exchange of ethanol instead of methanol could be considered an eco-friendly version of the original procedure, in addition to the time optimization to require just 1 day for the reaction to occur.

### 3.1 Physicochemical properties of AgMOFs

**FIGURE 1:**
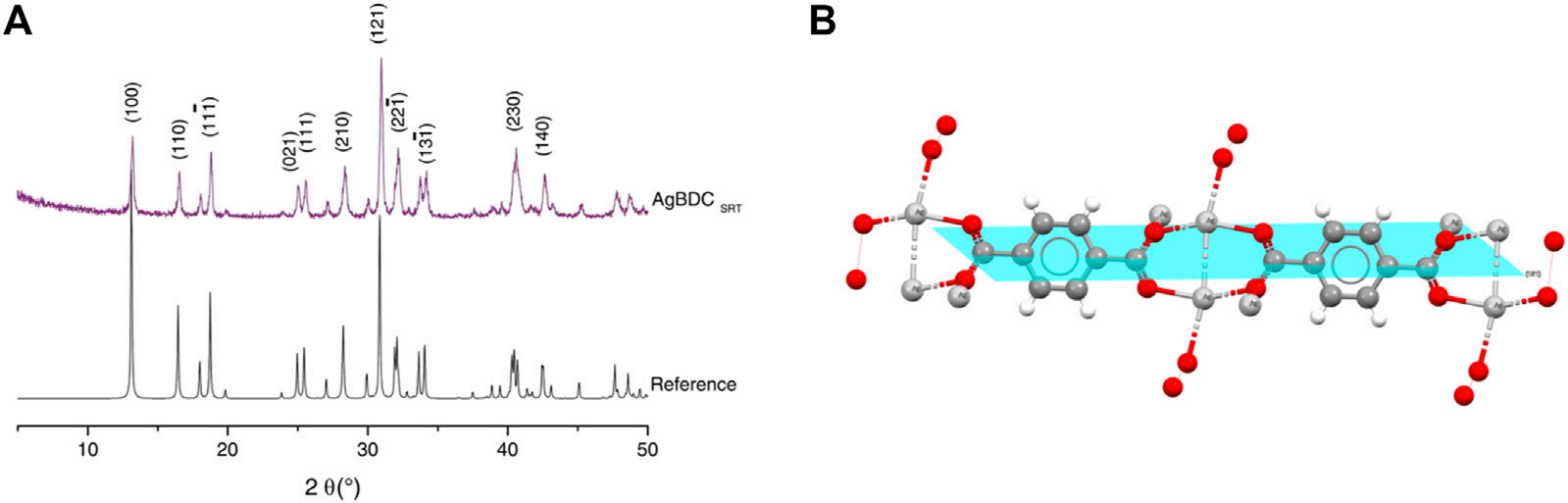
Crystallographic information for Ag_2_BDC. **(A)** XRD patterns. **(B)** Preferential growth plane [1 2 1].

**FIGURE 2:**
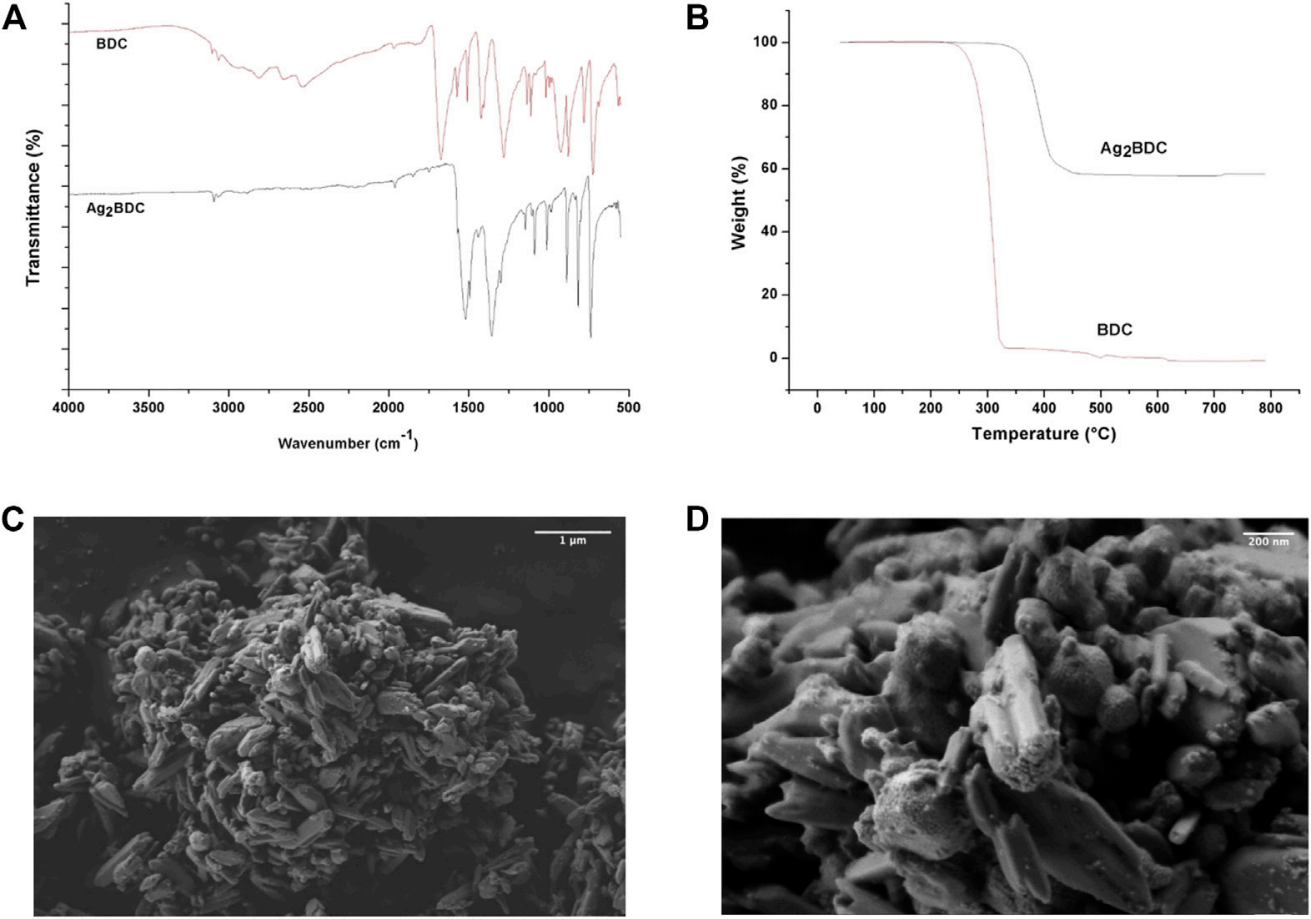
Physicochemical properties of MOF Ag_2_BDC. **(A)** TGA. **(B)** FT-IR. **(C)**, **(D)** SEM micrographs.

**Ag**_**2**_ **BDC:** The XRD pattern of **Ag**_**2**_ **BDC** showed the same diffraction peaks as the theoretical pattern (Figure 1A) (Sun et al., 2004). Thus, the same phase of the **Ag**_**2**_ **BDC** framework, with P 2_1_/c monoclinic system was obtained but with a shortened procedure lasting just 24 h. **Ag**_**2**_ **BDC** presented a crystalline structure with preferential growth in the planes located at position 2θ [°]: 30.9 [1 2 1], very to the preferential growth observed for UiO-66(Zr) in a modulated synthesis (Shan et al., 2018). This particular plane (Figure 1B) grew faster in the material due to the use of the ethanol cosolvent. The analysis of this particular plane showed that this solvent accelerated this growth direction growth due to better solubilization of the BDC ligand. In the [1 2 1] plane, the calculated crystal size (D) in the peak with the highest intensity was around 68.0 nm in at least one dimension of the material. This was corroborated by SEM (Figure 2), in which the **Ag**_**2**_ **BDC** crystals also achieved a nanometric-sized version, as described in the following paragraphs.

The FT-IR is shown in Figure 2A. The bands between 670 and 1228 cm^−1^ corresponded to the bending of C-H groups on the aromatic ring. The bands located between 1492 and 1529 cm^−1^ were attributed to vibrations from the interactions of metals with carboxylates in **Ag**_**2**_ **BDC**. These are related to asymmetric stretching between *aniso*- and *iso*-bidentate coordination modes for the -COO group that interacts with three and two Ag atoms, the first in a T-shaped coordination mode, and the latter in a more common bidentate fashion (Zhang et al., 2009; Guo et al., 2017). The T-shaped coordination mode developed a lower bond order compared to that only interacting with two Ag atoms, regardless of whether it was *iso*- or *aniso*-bidentate. This occurs due to the interaction with three Ag atoms instead of only two, which caused a decrease in the observed stretching frequency for C=O. Additionally, the literature has reported that the stretching bands at 758 cm^−1^ could be associated with the Ag-O bond, while the bands at 528 and 454 cm^−1^ are the bending vibrations of the parent O-Ag-O moiety (Zhang et al., 2009; Guo et al., 2017). These vibrations were slightly shifted at 740, 530, and 448 cm^−1^ in **Ag**_**2**_ **BDC** material, the former lower frequencies due to the presence of a slightly weakened Ag-O bonding, which crucially resulted in the absence of O-H stretching bands of atmospheric water, reaction media solvent, and COOH of BDC ligands in the FT-IR spectrum of **Ag**_**2**_ **BDC** (Figure 2A). The spectrum was also compared to that of free BDC ligand. No bands belonging to the -OH groups were present, which are regularly located between 3000 and 3500 cm^−1^ (Coates, 2000), nor the C=O of the free BDC ligand that normally appears at 1674 cm^−1^ (Figure 2A). This important result indicated the closed-packed nature of **Ag**_**2**_ **BDC** and the absence of guest molecules in the net, which was reinforced by the TGA findings described in the following paragraphs.

Figure 2B shows the TGA of this material, with weight loss observed as a plateau between room temperature and 330°C, indicating that no reaction solvent, humidity, or remnant BDC organic ligand molecules were occluded nor entrapped in this MOF. The absence of these compounds was initially ruled out by FT-IR (Figure 2A). Residual material corresponding to 58% of the original weight was observed for **Ag**_**2**_ **BDC** (theoretical = 56.7%, ∆ = −1.3%), which was mainly associated with residual silver (Ag^0^) and carbon ashes. This latter was determined by XRD of these remnants since the reflections that occur corresponded to the characteristic peaks of silver, indexed using the JCPDS card (00-0004-0783). Thus, no silver oxides were observed in this sample. These results may be due to the Ag-Ag self-association in the initial compound, the higher proportions of silver in the material because of the thermal sweep, and the almost complete degradation of the BDC organic linker (∆ = −1.3%).

The SEM micrographs of this material at different magnifications are shown in Figures 2C, D. The MOF showed agglomerating particles. A closer approach showed that these particles comprised various rods, including single rods *ca*. 600 nm in length, all of which showed poorly defined faces. Other morphologies included those that resembled sheet stacking with poorly defined faces of about 600 nm, as corroborated by XRD, with preferential growth at the [1 2 1] plane. The 50,000 kX magnification showed oval particles with poorly defined faces between 100 and 500 nm, with parallel agglomerations of rods. In the perpendicular direction, the rods showed rough regions. Additionally, on some faces, the adhesion of small particles (∼40–10 nm) to the surface was observed.

**FIGURE 3:**
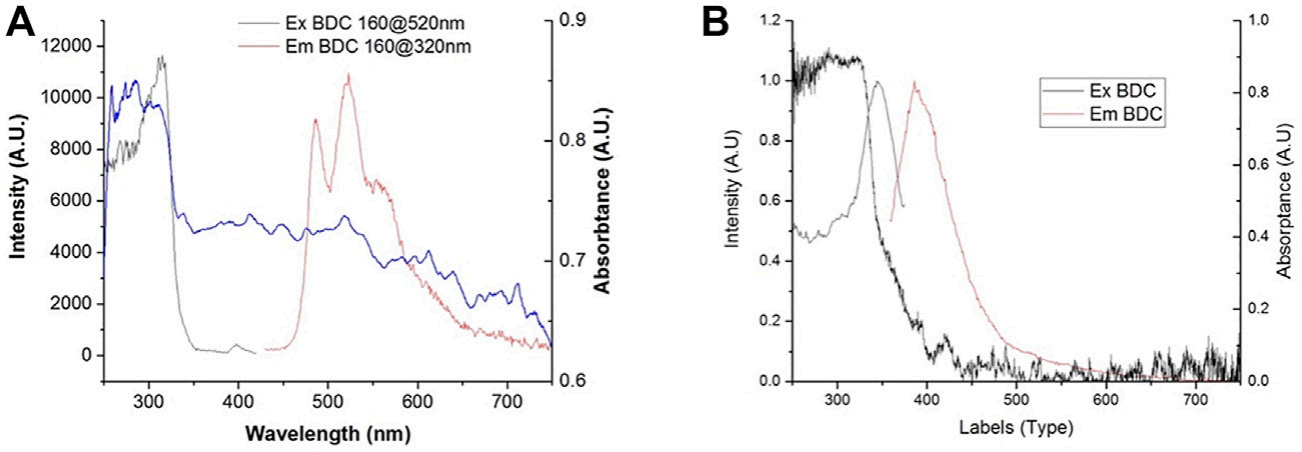
Absorption, excitation, and emission spectra of the **(A)** Ag_2_BDC MOF and **(B)** BDC ligand.

**Ag**_**2**_ **BDC** showed an average optical absorptance of >70% in the visible region, which increased to >80% at wavelengths of <330 nm, Figure 3A. Compared to the absorptance of the BDC ligand in Figure 3B, the high-absorption region is also observed at wavelengths <330 nm. Thus, the absorption in this region could be attributed to the presence of the ligand in the material. The radiation absorption at such wavelengths was attributed to π–π* transitions of the benzene ring.

The luminescence results of the BDC ligand showed an emission band peaking at 390 nm, which was excited mainly by wavelengths at the edge of the absorption band, with a maximum intensity at 347 nm, Figure 3B. The emission at 390 nm attributed to the ligand was not present in the emission spectrum of **Ag**_**2**_ **BDC**; instead, the observed emission corresponded to that previously reported for this compound (Gutiérrez et al., 2020), with three maxima at 496, 520, and 560 nm (Figure 3A).

The excitation spectrum of this MOF showed a maximum at 330 nm. The occurrence of this maximum in the high-absorbance region of the ligand indicates that the ligand absorbs the energy and, instead of presenting its natural emission at 390 nm, it presumably performs a ligand-to-metal energy transfer. Liu et al. (2008) and Gutiérrez et al. (2020) both attributed the emissions at 520 and 560 nm to a ligand-to-metal charge transfer, which is typical for d^10^ ions linked to oxygen, where an electron in a molecular orbital of the Ag-O bond is excited to an antibonding state of the Ag^+1^ ion, from which it decays radiatively (Blasse and Grabmaier, 1994). However, while Ying-Ying et al. attributed the emission at 496 nm to a remnant of a ligand-to-ligand energy transfer, Gutierrez et al. attributed it to a ligand-to-metal or ligand-to-metal–metal charge transfer. Based on the distance between ligands in orthogonal-plane C-H··· π = 2.724 Å, the interaction present for π_BDC_···Ag = 2.891 Å, and the lack of BTC···BTC coplanar interaction, all obtained from the single crystal X-ray diffraction structure, it would be hard for a ligand-to-ligand energy transfer to occur due to the low overlap of the π electronic clouds of the aromatic rings. Additionally, the organic ligand does not produce an emission at 496 nm. Thus, this emission cannot be attributed to a remnant of a ligand-to-ligand emission process; instead, it must be due to a ligand-to-metal (O_BTC_-Ag) or a ligand-to-metal–metal (O_BTC_-Ag-Ag) charge transfer.

#### 3.1.1 UAM-1

**FIGURE 4:**
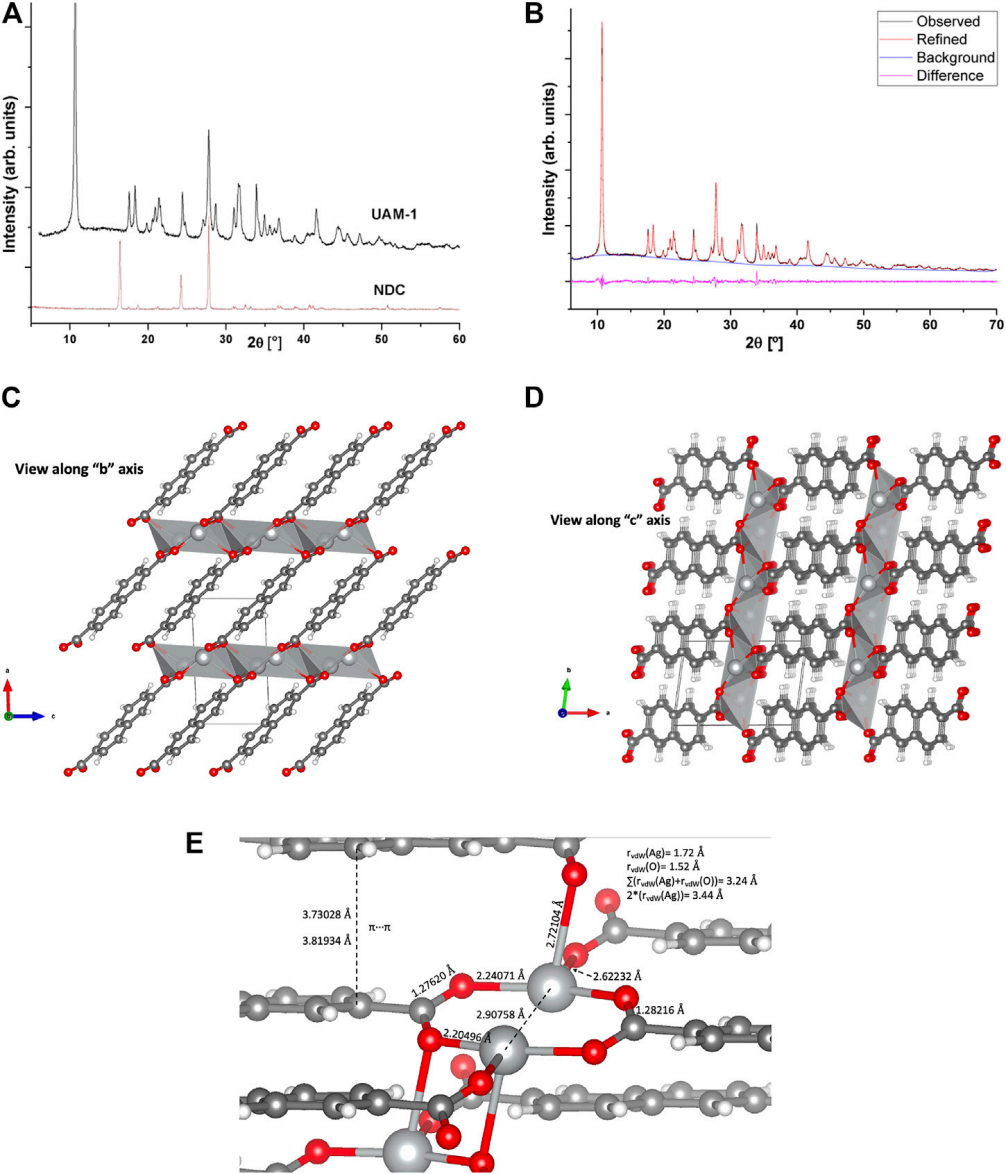
Crystallographic information for UAM-1. **(A)** XRD (UAM-1, top; NDC, bottom). **(B)** Experimental XRD, top; Rietveld-refined XRD, middle; XRD difference, bottom. **(C,D)** UAM-1 refined structure along the “b” and “c” axes, respectively. **(E)** UAM-1 SBU bonding scheme details.

The XRD pattern of **UAM-1** is shown in Figure 4A. This structure is very similar to that of the **Ag**_**2**_ **BDC** framework but with a *P -1* triclinic crystalline system. This structure was obtained by powder X-ray diffraction, DFT-quantum chemical calculations, and Rietveld mixed refinement (Figure 4B). Table 1 shows the most important XRD data for **UAM-1**. PXRD (2θ, º, [h k l]) was the most predominant according to intensity (>2500 counts), at 10.64 [1 0 0], 17.53 [1 1 0], 18.31 [0 0 1], 21.39 [2 0 0], 21.60 [1 0 1], 21.91 [1 1 -1], 24.40 [1 -1 1], 27.79 [2 0 -1], 27.83 [2 1 -1], 31.59 [0 2 -1], 31.62 [2 -1 1], 31.66 [2 -1 1], 31.75 [1 2 -1], and 33.91 [2 1 1].

**TABLE 1:** Crystallographic data for UAM-1.

	DFT and Rietveld refinement*
Chemical formula	C₁₂H₆O₄Ag₂
Formula weight, g·mol^−1^	429.91
Crystal system	Triclinic
Space group	*P-1*
a [Å]	8.39903
b [Å]	6.04421
c [Å]	5.03306
α [°]	105.965
β [°]	94.300
γ [°]	81.821
Volume [Å^3^]	242.98
Z	2^a^
T [K]	298.15
Number of reflections	210
ρ_calc_ [g·cm^−3^]	5.88
2θ range [°]	6.000–70.000
2θ step [°]	.02
R indexes [all data] [%]	R_P_ = 2.429; R_PW_ = 3.149
λ(Cu_Kα_) [Å]	1.54056

Weighting scheme: w = 1·counts^−1^.

^a^ Only half of the molecular structure appeared in the asymmetric unit.

SBU in **UAM-1** is a 2D (Ag-O)_n_ sheet, where NDC ligands interlay up and down to form a 3D architecture (Figures 4C,D). In comparison, three reported Ag-coordination polymers [Ag (fbc)]_n_, [Ag_2_ (cpd)]_n_ and [Ag_2_ (idc)]_n_ (fbc, 4-fluorobenzoic acid; cpd, cyclopentane-1,1-dicarboxylic acid; idc, iminodiacetic acid) developed very similar coordination environments (Zhu et al., 2003). These materials showed Ag···Ag distances ranging from 2.850, 2.789, 2.850, 2.939, and 2.996 Å (Figure 4E), all of which were <2 times the van der Waals radii of Ag (2*r_vdW_ = 3.44 Å) and very close to the Ag···Ag distance in metallic silver (2.89 Å), clearly indicating a strong Ag···Ag interaction (Zhu et al., 2003). In **UAM-1**, the Ag···Ag distance was 2.90758 Å, with four Ag-O bond distances (2.20496, 2.24071, 2.62232, and 2.72104 Å). Given the sum of vdW radii for Ag and O of 3.24 Å, all of the aforementioned distances were below the r_vdW_ sum for both atoms, thus assuring the nature of the Ag-O bonding. As the former two correspond to the *iso*bidentate coordination mode of the COO moiety to two silver atoms, this was also evidenced by the C⎓O pseudo double bonding in both parts of the COO moiety, at 1.276 and 1.282 Å, which are <.5% of the length difference of each other to determine the iso-coordination scheme. The third Ag-O bond corresponded to that developing the 2D sheets of the material, is of T-shape according to carboxylate, and is the bond that caused the slight elongation of the parent C=O bond. Finally, the fourth and longest Ag-O bond interlinked the 2D layers to develop the 3D structure. This 2D-layer interlinking also provoked a COO rotation, as evidenced by the dihedral angle between COO and aromatic naphthalene moiety of 26.79° (the same fragment for **Ag**_**2**_ **BDC** has a 22.33° angle to interlink a ladder structure) (Sun et al., 2004). Additional interesting structural feature are the π···π equilibrium distances of 3.73028 and 3.81934 Å for the two closest atoms among the aromatics. Compared to the previously reported **Ag**_**2**_ **BDC** structure (Sun et al., 2004), the **UAM-1** structure preserves some bonding scheme similarities; for e.g., the four Ag-O bond distances (Å) are comparatively similar (**Ag**_**2**_ **BDC**/**UAM-1:** 2.208/2.20496, 2.230/2.24071, 2.511/2.62232, and 2.511/2.72104) and to [Ag (fbc)]_n_, [Ag_2_ (cpd)]_n_ and [Ag_2_ (idc)]_n_. The **Ag**_**2**_ **BDC** structure develops a ladder interlinking scheme, while the **UAM-1** develops an interlinked 2D sheet. This difference could be due to the enhanced aromatic moiety in NDC that requires π···π interactions, which are not present in **Ag**_**2**_ **BDC**.

**FIGURE 5:**
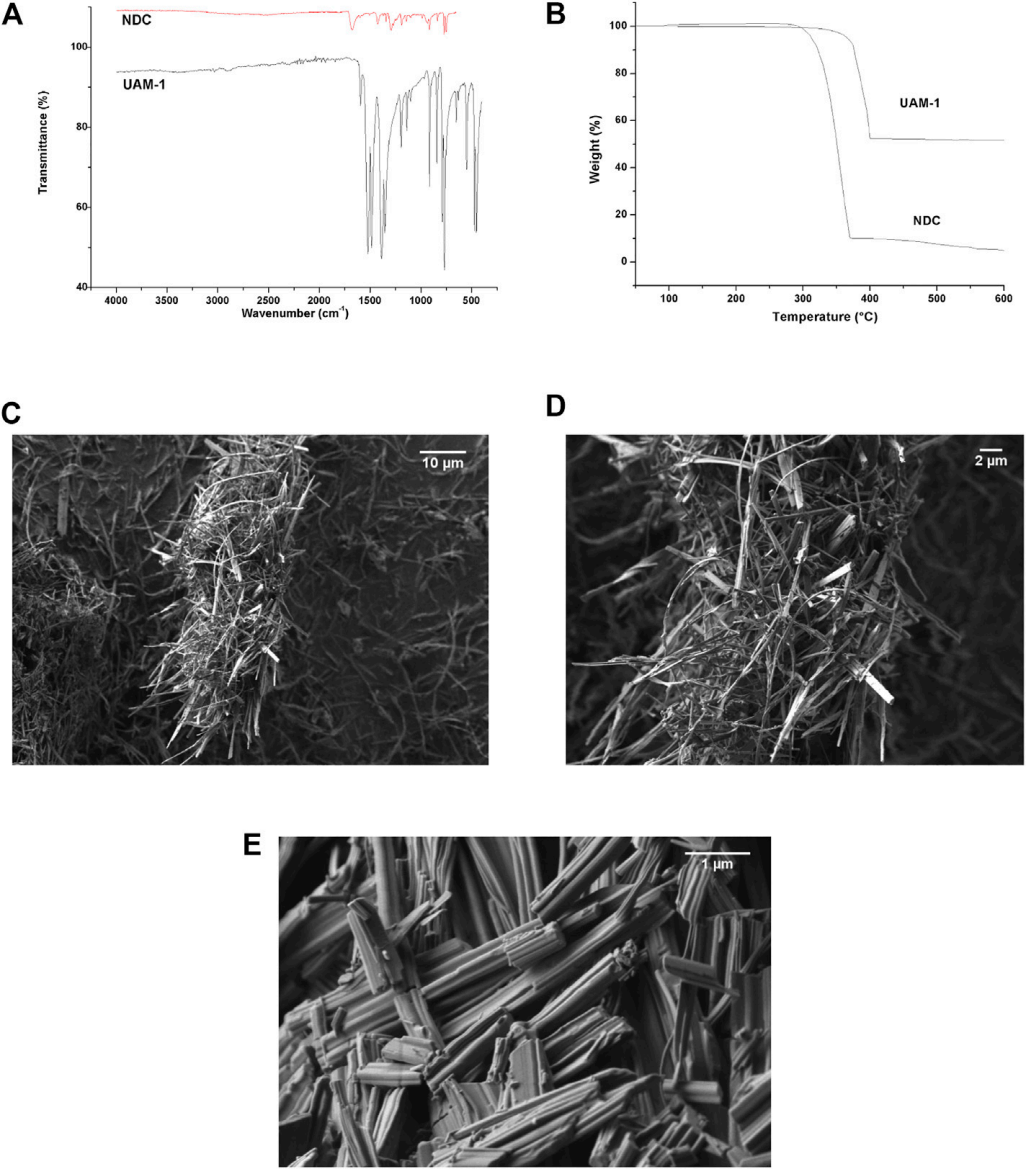
Physicochemical properties of UAM-1. **(A)** TGA. **(B)** FT-IR. **(C)**, **(D)**, **(E)** SEM micrographs.

The FT-IR is shown in Figure 5A. The bands at 632, 653, 789, 842, 916, 1101, 1138, and 1195 cm^−1^ correspond to the bending of C-H groups belonging to the aromatic moiety. The bands at 3063 and 3032 cm^−1^ correspond to the stretching of C_Ar_-H of the NDC ligand. The bands located between 1487, 1523, and 1595 cm^−1^ could be attributed to vibrations from the interaction of metals with carboxylates in **UAM-1**. These reflect the stretching of *aniso*- and *iso*-bidentate coordination modes for the -COO group interacting with three and two Ag atoms, the first in a T-shaped coordination mode, and the second in a bidentate fashion, very similar to **Ag**_**2**_ **BDC** (Zhang et al., 2009; Guo et al., 2017). Meanwhile, the band at 1595 cm^−1^ occurs due to COOAg metallic esters not present in **Ag**_**2**_ **BDC**. Finally, the band at 769 cm^−1^ corresponds to the Ag-O bond, while those at 548, 473, and 453 cm^−1^ correspond to the bending of the O-Ag-O moiety (Zhang et al., 2009; Guo et al., 2017). As in **Ag**_**2**_ **BDC**, these resulted in a lack of O-H stretching bands from atmospheric water, the reaction media solvent, and the COOH of the NDC ligand in the FT-IR spectrum (Figure 5A). This evidence also demonstrated the closed-packed nature of **UAM-1** and the absence of guest molecules into the net, reinforced by the TGA finding that is described later. Additional evidence is the BET area of the material (m^2^·g^−1^) of 11.51.

Figure 5B shows the TGA of this material, in which weight loss was observed between room temperature and 310°C, again (as in **Ag**_**2**_ **BDC**) indicating that no reaction solvent, humidity, or remnant NDC organic ligand molecules were occluded nor entrapped in this MOF. The absence of these compounds was clearly indicated by FT-IR (Figure 5A). Residual material corresponding to 57% of the original weight was shown for **UAM-1**, which was mainly associated with residual silver (Ag^0^) and carbon ashes (Supplementary Figure S2).

The micrographs for this material at different magnifications are shown in Figures 5C–E. The **UAM-1** MOF developed agglomerating fibrillar particles >10 μm. These fibrils showed widths varying from approximately 500 to 50 nm. The 15,000 kX magnification (Figure 5E) showed extruded fibrils with different amounts of gathered fibers.

#### 3.1.2 UAM-2

**FIGURE 6:**
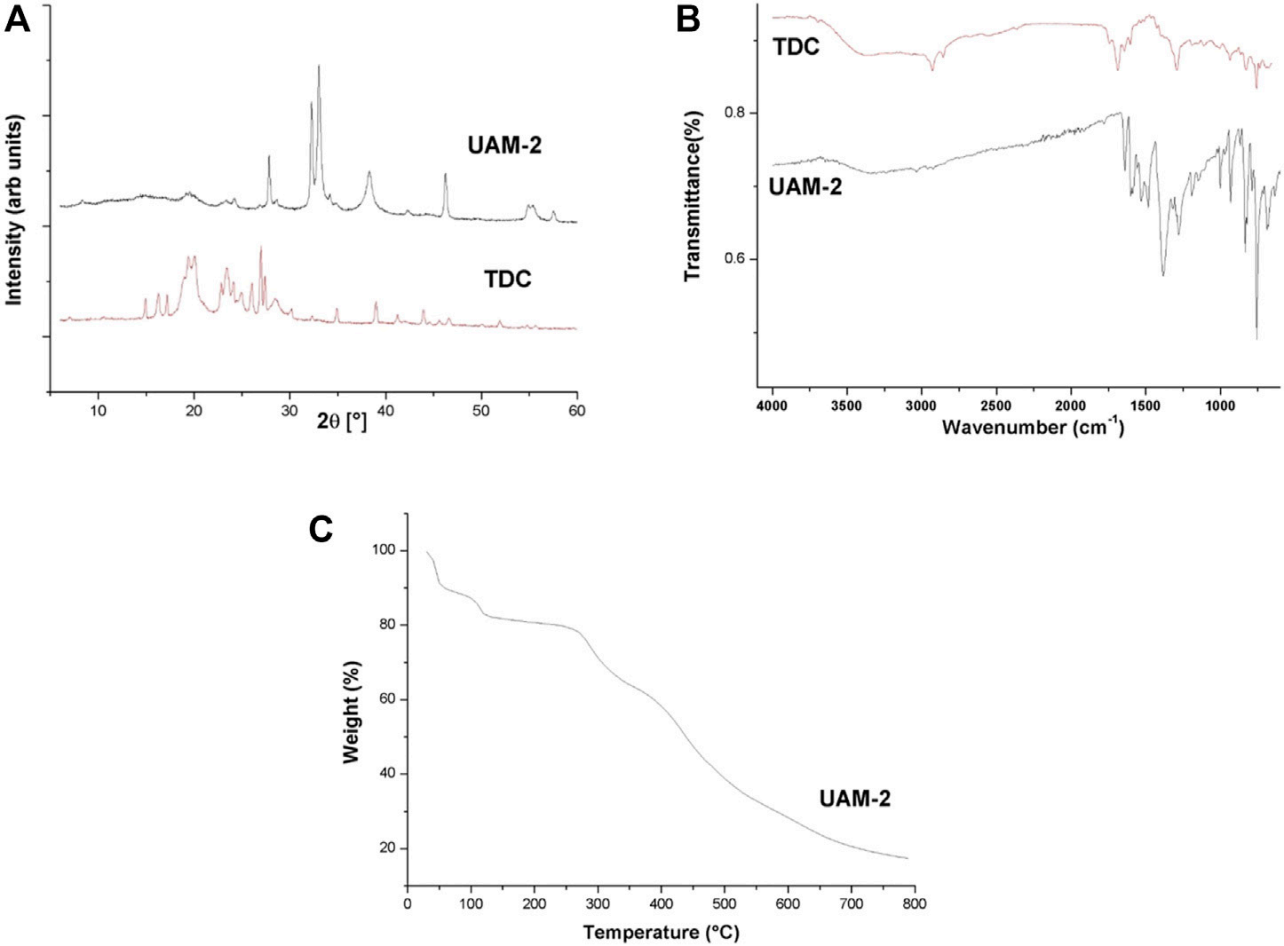
**(A)** XRD (UAM-2, top; TDC bottom). **(B)** FT-IR (UAM-2, bottom; TDC, top). **(C)** TGA.

The XRD pattern of **UAM-2** is shown in Figure 6A and differs from that of the TDC ligand. The diffractogram shows five high-intensity peaks at (2θ [°], (intensity)) 27.84 (7642), 32.26 (12515), 33.03 (15834), 38.30 (6201), and 46.28 (5996), which define the highly crystalline nature of the sample.

The FT-IR of this material is shown in Figure 6B, with the bands at 636, 687, 767, 834, 867, 932, 1101, 1148, and 1191 cm^−1^ corresponding to C-H bends at the aromatic moiety. The band at 3028 corresponds to the stretching of the C_Ar_-H of the TDC ligand. The bands at 1483, 1529, 1542, 1587, 1598, and 1639 cm^−1^ could be attributed to vibrations from the interaction of metals with carboxylates in **UAM-2**. These are also (as in **Ag**_**2**_ **BDC** and **UAM-1**) stretching of *aniso*- and *iso*-bidentate coordination modes for the -COO group interacting with three and two Ag atoms, the first in a T-shaped coordination mode, and the second and third in a bidentate fashion, very similar to **Ag**_**2**_ **BDC/UAM-1** (Zhang et al., 2009; Guo et al., 2017). Meanwhile, the bands at 1587 and 1598 cm^−1^ are due to COOAg metallic esters present only in **UAM-1**. The bands at 767 & 756 cm^−1^ correspond to the Ag-O bond, while the bands at 538 and 463 cm^−1^ reflect the bending of the O-Ag-O moiety (Zhang et al., 2009; Guo et al., 2017). In contrast to the previous two materials, our results clearly demonstrate the presence of O-H stretching bands in atmospheric water or reaction media (consistent with the TGA findings) solvent, with no evidence of the COOH of the TDC ligand in the FT-IR spectrum (Figure 6B). Nevertheless, regarding these differential experimental findings, the BET area of the material (m^2^·g^−1^) was very low in this case (6.97).

Figure 6C shows the TGA of **UAM-2**. This is the only structure to show a weight loss of ca. 18% at low temperatures (<130°C), providing insights into the presence of solvent molecules, as also observed in the FT-IR. Moreover, a plateau around 130 to 250°C could be considered the stability interval of the material. Beyond this are two clear decompositions, the first ranging from 350–470°C, in which 18.60% of the material is lost, and the second at 470–800, corresponding to a 26.23% loss. Finally, 19.15% of the material remained as ashes, in this case mostly those of Ag⁰.

### 3.2 Luminescence in the UAM-1 and UAM-2 structures

**FIGURE 7:**
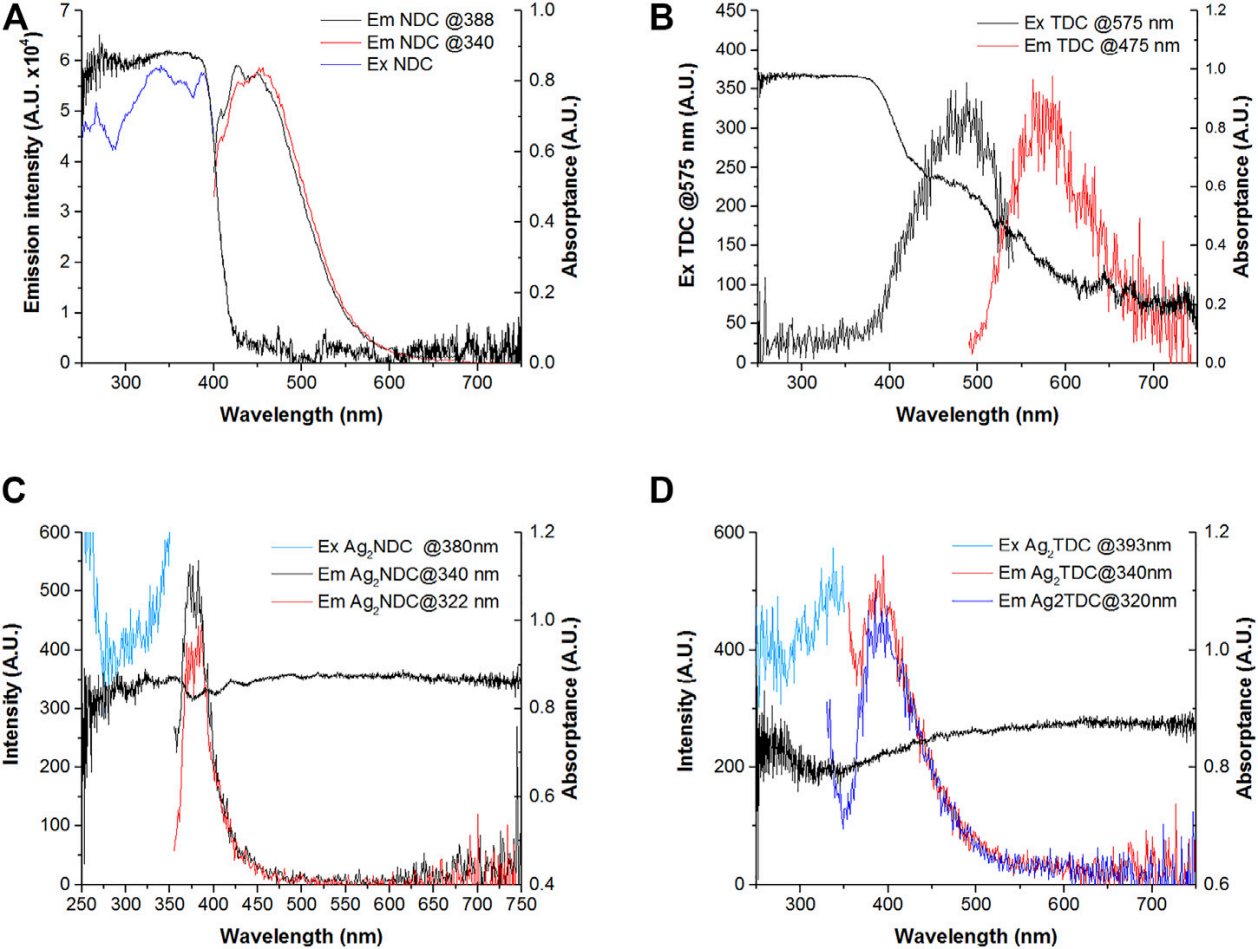
Absorption, excitation, and emission spectra of **(A)** NDC. **(B)** TDC. **(C)** UAM-1. **(D)** UAM-2.

The luminescent emission of the NDC ligand, with a broad emission band between 400 and 550 nm peaking at 472 nm and excitation at 340 nm, reproduces that reported by Bing An et al., who excited samples with the same wavelength (An et al., 2014). This emission is attributed to π*–π or π*–n intramolecular transitions and presents an excitation band extending along the strong absorption band below 400 nm (Figure 7A). In the present study, the NDC sample showed high absorption in the visible region, from 400 to 600 nm, which increased at wavelengths <400 nm. According to the excitation spectra, the absorption region between 400 and 600 nm promoted luminescence in this molecule, which consists of a broad band peaking at 587 nm. The absorption at short wavelengths might be attributed to π–π* or n–π* at the aromatic rings, while the absorption at larger wavelengths might be attributed to transitions between aromatic rings, where the delocalization of π electrons might decrease the energy difference between molecular orbitals, producing an emission at even lower photon energies.

Unfortunately, no emission for the pure TDC ligand has been reported. The reported studies usually included different organic substituents and functional groups along the terphenyl core, including 5′-carboxyl-[1,1′:3′,1″-terphenyl]-4,4″-dicarboxylic acid (Li et al., 2012). The emission reported by Liming Fan, et al., which is the closest, used p-terphenyl-2,2″,5″,5‴-tetracarboxylic acid, which showed a broad band from 350 to 550 nm, peaking at 410 nm (Figure 7B) (Fan et al., 2019).

The **UAM-1** and **UAM-2** compounds showed significant changes in optical absorption, with absorptance values >80% in the visible region, consistent with the black colors of the samples (Figures 7C and 7D). The emission in both samples differed from those of the free ligands in both bath position and width. **UAM-1** presented a narrower band from 350 to 400 nm, peaking at 380 nm, while **UAM-2** presented a band from 350 to 500 nm, peaking at 393 nm. Based on the structures obtained by Rietveld analysis and refinement, the distance between the naphthalene···naphthalene and terphenyl···terphenyl moieties was <5 Å, suggesting an overlap of electronic clouds and electronic transitions between aromatic moieties that might be responsible for the emission.

### 3.3 AgMOF persistency

One of the great challenges of using MOF architectures in aqueous media is their low or practically no stability (Wang et al., 2016; Feng et al., 2018; Zhang et al., 2020). To determine the stability of the obtained AgMOFs, this study performed tests with deionized water and different physiological and culture media used in biological tests. In most cases, stability is essential for MOFs to retain their properties and characteristics, if necessary, and depends on the ligand type and nature, the metal ion, the coordination energy between metal and ligand, the structure, the conditions in which the network is exposed, and the number of defects in the structure (Yuan et al., 2018). Likewise, the degradation of materials in culture media could be a strategy for the gradual release of biologically active molecules or even elements/atoms/ions or fragments comprising the network for biological applications. The tests were performed at three different temperatures, 4°C, 37°C, and room temperature, to evaluate their effects on the stability. However, as the results were very similar, only those obtained at 37°C were presented. Moreover, since this is the physiologic temperature, it is a reference for materials in biological applications.

Supplementary Figure S3 shows the diffractograms of materials in contact with deionized water for 24 h compared to the reference diffractograms of each material. The structure and crystallinity of the MOFs were maintained after contact with water. Subsequently, the materials were evaluated in PBS, DMEM, and DMEM+FBS, at 37°C for 24 h (Ruyra et al., 2015). Supplementary Figure S4 shows the subsequent MOF patterns, in which the materials lost their initial crystalline structure in all media. The reflections shown in Supplementary Figure S4 corresponded to the characteristic peaks of silver chloride (AgCl), indexed using the JCPDS card (98-006-0414). The media are rich in ions; however, soft–soft (Pearson’s principle) affinity between silver and chlorine is evident in these results, where the soft acid (Ag) preferred to react with the soft base (Cl), which resulted in the disassembly of the starting material. This effect caused structure breakdown and formation of the AgCl compound, which could be a strategy for the degradation of these materials in due course.

## 4 Conclusion

This study synthesized three silver MOFs by a modified methodology providing moderate yields within 1 day or reaction, compared to previous methodologies that yielded the same amounts of material but required 1 week under solvothermal or reflux conditions. This new synthetic methodology was applied to previously reported **Ag₂BDC** material but also was extended to two new materials, **Ag₂NDC** (**UAM-1)** and **Ag₂TDC** (**UAM-2**). This synthesis also provided nanosized materials for two cases, shaping nanorods and layered assemblies for **Ag₂BDC**, and nanofibrils for the case of **UAM-1**. All of them were thoroughly characterized by powder XRD, FT-IR, TGA, and luminescence spectroscopy, which provided useful information for the correct identification of these materials. Additionally, the **UAM-1** structure was obtained by DFT periodic calculations and minimization by a structure solution model, starting from the **Ag₂BDC** and resolved through Rietveld refinement. This structure showed motifs similar to those for **Ag₂BDC**, mainly in the COO-Ag fragment of the material, showing two characteristic Ag···Ag interactions. The FT-IR in all cases revealed stretching bands (cm^−1^) located around 1450 and 1680, correlating to asymmetric stretching between aniso- and iso-bidentate coordination modes present at the -COO group that, in general, interact with three Ag atoms. Additionally, bands belonging to the Ag-O bonds of the materials around 780–740 were observed, providing evidence for the network coordination and formation. In particular, while the luminescence studies showed on one hand that the typical emission of **Ag₂BDC** sample was attributed to ligand-to-metal or ligand-to-metal–metal transitions, in this material, transference is present due to the short distance between adjacent BDC molecules in the material. On the other hand, **UAM-1** and **UAM-2** showed no luminescence emission attributable to a ligand-to-metal transition, presenting only emissions in the UV region due to a ligand-to-ligand transition, promoted by electronic clouds of adjacent ligands at distances <5 Å. This latter finding provided insights into the importance of the material structure for the development of such luminescent properties for plausible applications. Finally, the stability of **Ag₂BDC** and **UAM-1** was assessed in PBS, DMEM, and DMEM+FBS, at 37°C for 24 h. The XRD from the remnants demonstrated that tested materials lost their crystalline structure in these media and remained in the water. The reflections in the resulting diffractograms corresponded to silver chloride (AgCl) peaks, showing that the soft–soft affinity between silver and chlorine, according to Pearson’s principle, led to the disassembly of the starting material.

## Data Availability

The original contributions presented in the study are included in the article/Supplementary Material; further inquiries can be directed to the corresponding authors.
