# Supplementary material for: Room-temperature synthesis of nanometric and luminescent silver-MOFs
Source: Front Chem. 2023 Jan 6;10:1065622. doi: 10.3389/fchem.2022.1065622 (PMC9853072; doi:10.3389/fchem.2022.1065622)
Supplement: Supplementary file 1 [file DataSheet1.docx]

**Supporting Information**

**Room temperature synthesis of nanometric & luminescent silver-MOFs**

Vanessa Celis-Arias, Ismael A. Garduño-Wilchis, Gilberto Alarcón, Fernando González Chávez, Efrain Garrido Guerrero, Hiram I. Beltrán,^*^ Sandra Loera-Serna^*^

a) b)

c)

Figure S1. a) Schematic representation of synthesis condition of **Ag_2_BDC** MOF, b) X-ray patters of molar ratio 2:1 Ag:BDC with different condition methods: SRT (stirring at room temperature), US (ultrasound assisted method) and, ST (solvothermal methodology), the red oval corresponds to Ag-NP diffraction peak and, c) XRD patter of **Ag_2_BDC** reproducibility test with STR method.


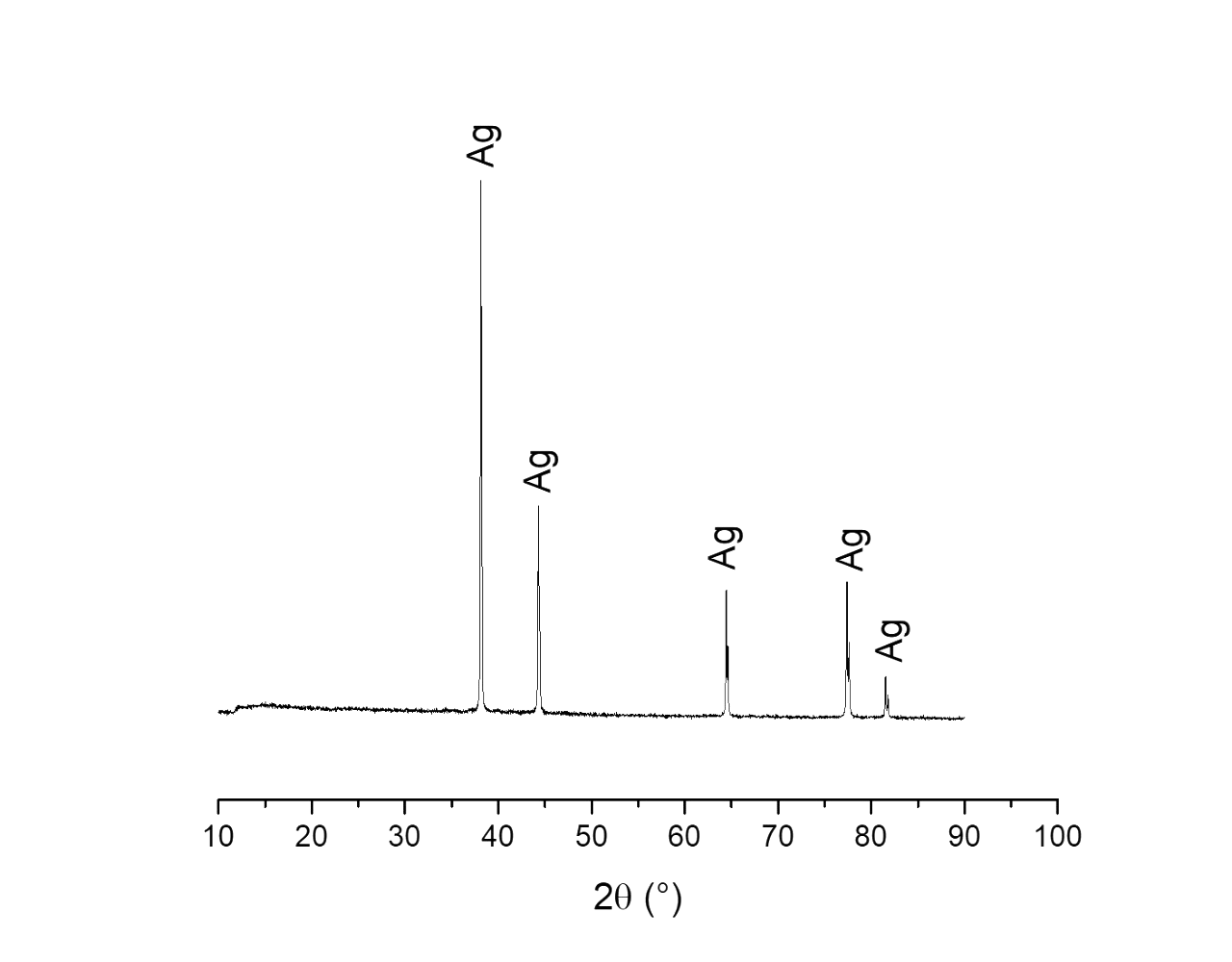


Figure S2. XRD of **Ag_2_BDC** remaining after TGA (800 ºC).

| 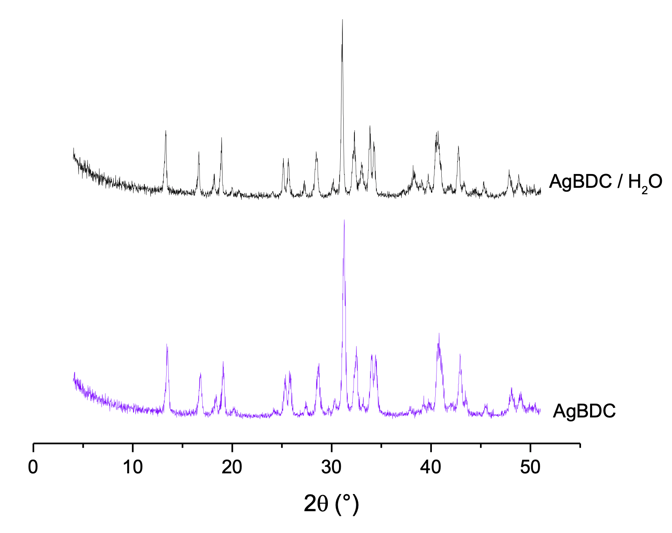 |
| --- |

Figure S3. Structural stability (24 h) of MOF **Ag_2_BDC** up) in water, down) as synthesized.

| 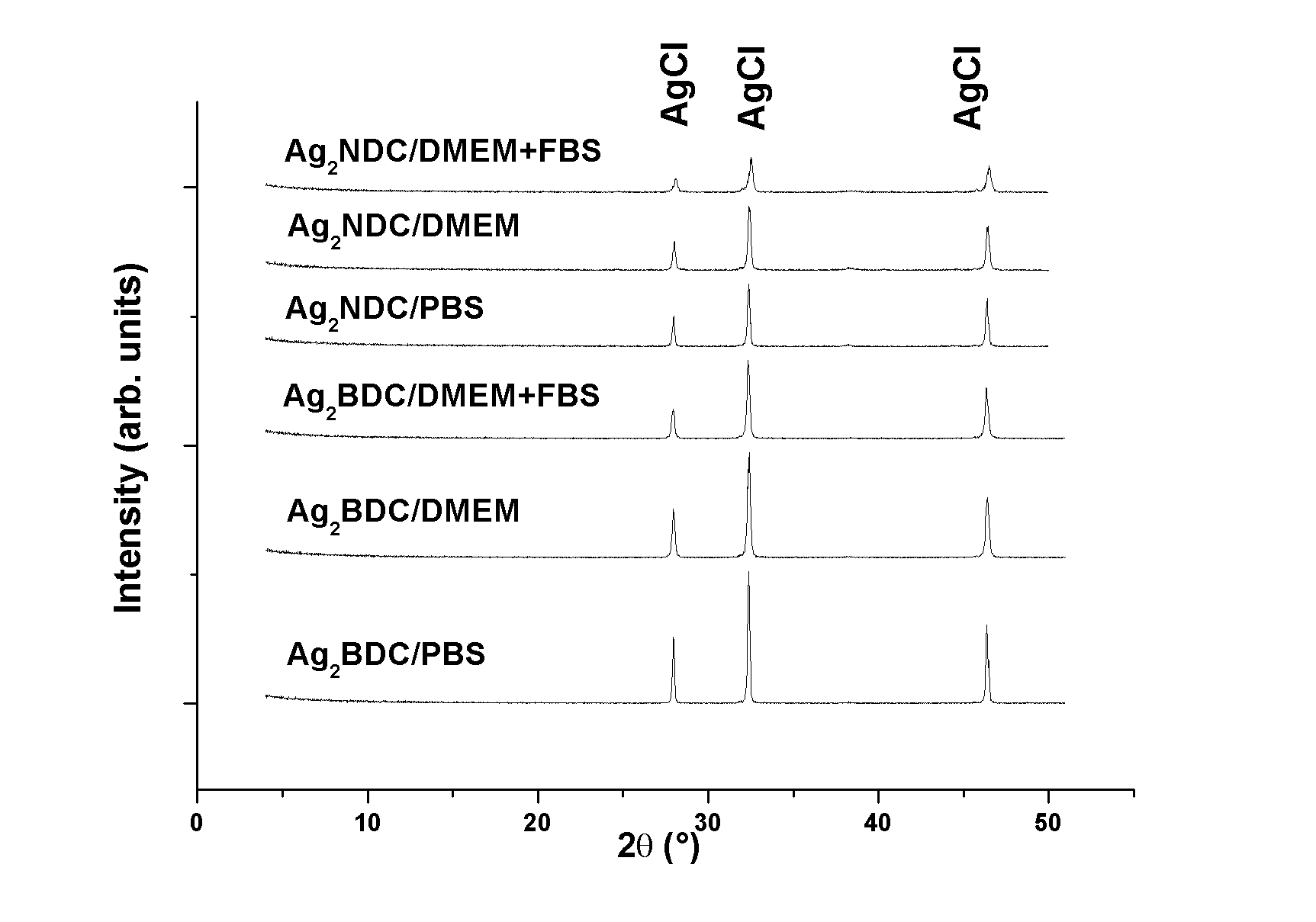 |
| --- |

Figure S4. Structural stability of MOFs **Ag_2_BDC** & **UAM-1** in PBS, DMEM, and DMEM+FBS. Herein is clearly evidenced the formation of AgCl in all tested cases.

XRD Structural data information of **UAM-1** (.cif file without structure factors).

Structure factors were not obtained since this resolution was determined by crystallographic cell enhancement, followed by ligand replacement.

#================================================================

data_Ag2NDC-UAM-1

#================================================================

_publ_section_title

;

Room temperature synthesis of nanometric & luminescent silver-MOFs

;

_publ_contact_author_name

;

Sandra Loera-Serna

Hiram Isaac Beltran

;

_publ_contact_author_address

;

Departamento de Ciencias B\'asicas, DCBI, Universidad Aut\'onoma

Metropolitana-Azcapotzalco, Av. San Pablo No 180, Col. Reynosa-Tamaulipas,

Ciudad de M\'exico. C.P. 02200, M\'exico.

;

_publ_contact_author_email

;

sls@azc.uam.mx

hibc@azc.uam.mx

;

_publ_section_abstract

;

Three silver-MOFs were prepared by an optimized, room-temperature methodology starting from AgNO₃ and dicarboxylate ligands in water/ethanol yielding Ag2BDC, Ag2NDC (UAM-1) & Ag2TDC (UAM-2) in 38-48% (BDC=benzenedicarboxylate; NDC=1,8-naphthalene-dicarboxylate; TDC=p-terphenyl-4,4′′-dicarboxylate). They were characterized by PXRD/FT-IR/TGA/photoluminescence spectroscopy, and the former two by SEM. These materials started decomposing at 330ºC evidencing stability. The crystal structure for UAM-1 was determined by PXRD, DFT calculations, and Rietveld refinement, in general, the structure is of 3D nature, where the largest Ag-O bonding is interlinking 2D-layers. FTIR spectra revealed 1450 and 1680 bands (cm-1) of asymmetric stretching aniso-/iso-bidentate -COO coordination with 2/3-Ag atoms, accompanied by Ag-O bands in 780-740, all evidenced the formation of the network. XRD and SEM, unveil nanometric-scale crystals of Ag₂BDC, and UAM-1 developed micrometric single-stranded/agglomerated fibrillar particles of varying nanometric widths. Luminescence spectroscopy presented emission of Ag₂BDC, attributed to ligand-to-metal or ligand-to-metal-metal transitions, evidencing an energy transfer due to a short distance between adjacent BDC molecules. UAM-1 and UAM-2 have not shown luminescence emission attributable to ligand-to-metal transition, presenting only emission in UV. Stability of Ag₂BDC and UAM-1 was evaluated in PBS/DMEM/DMEM+FBS media by XRD, losing their crystallinity, and yielding AgCl due to soft-soft (Pearson's principle) affinity.

;

_audit_creation_method Expo2014

_audit_creation_date 2022-08-16

_chemical_name_systematic Ag2NDC-UAM-1

_chemical_formula_moiety 'C12 H6 O4 Ag2'

_chemical_formula_sum 'C12 H6 O4 Ag2'

_chemical_formula_weight 429.91

_cell_formula_units_Z 2

loop_

_atom_type_symbol

_atom_type_description

_atom_type_scat_source

'H' 'Hydrogen' 'International Tables Vol C Tables 4.2.6.8 and 6.1.1.4'

'C' 'Carbon' 'International Tables Vol C Tables 4.2.6.8 and 6.1.1.4'

'O' 'Oxygen' 'International Tables Vol C Tables 4.2.6.8 and 6.1.1.4'

'Ag' 'Silver' 'International Tables Vol C Tables 4.2.6.8 and 6.1.1.4'

_cell_length_a 8.3990(6)

_cell_length_b 6.0442(3)

_cell_length_c 5.0331(3)

_cell_angle_alpha 105.965(5)

_cell_angle_beta 94.300(3)

_cell_angle_gamma 81.821(6)

_cell_volume 242.98(2)

_cell_measurement_temperature 298

_exptl_crystal_description polycrystalline

_diffrn_radiation_type 'Cu K\a~1~'

_diffrn_source 'Cu microsource'

_diffrn_measurement_device_type

;

'Bruker Advanced D8 diffractometer, LYNXEYE detector'

;

_computing_cell_refinement 'EXPO 2014 (Altomare 2014)'

_computing_structure_solution 'EXPO 2014 (Altomare 2014)'

_computing_structure_refinement 'EXPO 2014 (Altomare 2014)'

_diffrn_ambient_temperature 298

_diffrn_radiation_wavelength 1.540560

_symmetry_Int_Tables_number 2

_symmetry_cell_setting triclinic

_symmetry_space_group_name_H-M 'P -1'

_symmetry_space_group_name_hall '-P 1'

loop_

_symmetry_equiv_pos_site_id

_symmetry_equiv_pos_as_xyz

1 'x, y, z'

2 '-x, -y, -z'

loop_

_pd_proc_ls_profile_function pseudo-Voigt

_pd_proc_ls_background_function

;

Background modelled by a Chebyshev polynomial of degree 19

b1 3102(3)

b2 -691(3)

b3 29(3)

b4 127(3)

b5 -75(2)

b6 94(2)

b7 -1(3)

b8 -24(3)

b9 28(2)

b10 -14(2)

b11 31(2)

b12 -21(2)

b13 -8(2)

b14 -4(2)

b15 5(2)

b16 3(2)

b17 43(2)

b18 6.1(19)

b19 4.6(19)

zero 0.2218(5)

sample di.0.007538527

sample tr.-0.0001194932

scale 0.000901(7)

;

_pd_calc_method 'Rietveld Refinement'

#---- raw/calc data loop -----

_pd_meas_2theta_range_min 6.0000

_pd_meas_2theta_range_max 70.0000

_pd_meas_2theta_range_inc 0.0197

_pd_proc_2theta_range_min 6.0000

_pd_proc_2theta_range_max 70.0000

_pd_proc_2theta_range_inc 0.0197

_pd_meas_number_of_points 3255

_pd_proc_number_of_points 3255

_pd_proc_ls_prof_R_factor 0.02429

_pd_proc_ls_prof_wR_factor 0.03292

_pd_proc_ls_prof_wR_expected 0.02199

_refine_ls_R_Fsqd_factor 0.06622

_reflns_number_total 210

_reflns_limit_h_max 6

_reflns_limit_k_max 4

_reflns_limit_l_max 3

_reflns_d_resolution_high 1.34294

# ATOMIC COORDINATES AND DISPLACEMENT PARAMETERS

loop_

_atom_site_type_symbol

_atom_site_label

_atom_site_fract_x

_atom_site_fract_y

_atom_site_fract_z

_atom_site_U_iso_or_equiv

_atom_site_occupancy

_atom_site_adp_type

C C1 0.8779 1.2630 0.9249 0.0127 1.0000 Uiso

C C2 0.8779 1.2630 0.9249 0.0127 1.0000 Uiso

C C3 0.7783 1.1625 0.7069 0.0127 1.0000 Uiso

C C4 0.7783 1.1625 0.7069 0.0127 1.0000 Uiso

H H1 0.6914 1.2711 0.6098 0.0152 1.0000 Uiso

H H2 0.6914 1.2711 0.6098 0.0152 1.0000 Uiso

C C5 -0.0064 0.1240 0.0509 0.0127 1.0000 Uiso

C C6 -0.0064 0.1240 0.0509 0.0127 1.0000 Uiso

C C7 0.9000 0.7783 0.7317 0.0127 1.0000 Uiso

C C8 0.1000 0.2217 0.2683 0.0127 1.0000 Uiso

H H3 0.9052 0.5901 0.6570 0.0152 1.0000 Uiso

H H4 0.0948 0.4099 0.3430 0.0152 1.0000 Uiso

C C9 0.7869 0.9170 0.6136 0.0127 1.0000 Uiso

C C10 0.2131 0.0830 0.3864 0.0127 1.0000 Uiso

C C11 0.6729 0.7997 0.3960 0.0127 1.0000 Uiso

C C12 0.3271 0.2003 0.6040 0.0127 1.0000 Uiso

O O1 0.6127 0.8984 0.2110 0.0127 1.0000 Uiso

O O2 0.3873 0.1016 0.7890 0.0127 1.0000 Uiso

O O3 0.6452 0.6012 0.4137 0.0127 1.0000 Uiso

O O4 0.3548 0.3988 0.5863 0.0127 1.0000 Uiso

H H5 0.8674 1.4514 1.0037 0.0152 1.0000 Uiso

H H6 0.8674 1.4514 1.0037 0.0152 1.0000 Uiso

Ag Ag1 0.5337 0.3115 0.1316 0.0127 1.0000 Uiso

Ag Ag2 0.4663 0.6885 0.8684 0.0127 1.0000 Uiso

# MOLECULAR GEOMETRY

loop_

_geom_bond_atom_site_label_1

_geom_bond_atom_site_label_2

_geom_bond_distance

_geom_bond_site_symmetry_2

C1 C4 1.382933 .

C1 C6 1.421720 1_666

C1 H6 1.090919 .

C2 C3 1.382933 .

C2 C5 1.421722 1_666

C2 H5 1.090929 .

C3 H2 1.090781 .

C3 C10 1.420519 2_666

C4 H1 1.090792 .

C4 C9 1.420505 .

C5 C2 1.421722 1_444

C5 C6 1.435133 2_555

C5 C7 1.412118 2_666

C6 C1 1.421727 1_444

C6 C5 1.435133 2_555

C6 C8 1.412116 .

C7 C5 1.412117 2_666

C7 H4 1.090545 2_666

C7 C10 1.386132 2_666

C8 H3 1.090534 2_666

C8 C9 1.386139 2_666

H3 C8 1.090537 2_666

H4 C7 1.090548 2_666

C9 C8 1.386140 2_666

C9 C12 1.492947 2_666

C10 C4 1.420511 2_666

C10 C7 1.386130 2_666

C10 C11 1.492939 2_666

C11 C10 1.492936 2_666

C11 O2 1.275257 2_666

C11 O4 1.281428 2_666

C12 C9 1.492943 2_666

C12 O1 1.275276 2_666

C12 O3 1.281407 2_666

O1 C12 1.275274 2_666

O1 Ag1 2.240486 2_665

O1 Ag2 2.621906 2_676

O2 C11 1.275256 2_666

O2 Ag1 2.240485 1_556

O2 Ag2 2.621915 1_545

O3 C12 1.281406 2_666

O3 Ag1 2.720552 2_666

O3 Ag2 2.204131 2_666

O4 C11 1.281427 2_666

O4 Ag1 2.204118 2_666

O4 Ag2 2.720536 2_666

Ag1 O1 2.621920 1_545

Ag1 O1 2.240485 2_665

Ag1 O3 2.720548 2_666

Ag1 O4 2.204117 2_666

Ag2 O1 2.621908 2_676

Ag2 O2 2.240478 2_667

Ag2 O3 2.204129 2_666

Ag2 O4 2.720532 2_666

loop_

_geom_angle_atom_site_label_1

_geom_angle_atom_site_label_2

_geom_angle_atom_site_label_3

_geom_angle

_geom_angle_site_symmetry_1

_geom_angle_site_symmetry_3

C4 C1 H6 120.00 . .

C1 C4 H1 120.17 . .

C1 C4 C9 119.82 . .

C3 C2 H5 120.00 . .

C2 C3 H2 120.17 . .

H1 C4 C9 120.00 . .

C4 C9 C7 120.16 . .

C4 C9 C11 122.05 . .

C6 C8 H4 119.58 . .

C6 C8 C10 121.24 . .

H3 C7 C9 119.18 . .

C7 C9 C11 117.75 . .

H4 C8 C10 119.18 . .

C8 C10 C12 117.75 . .

C9 C11 O1 119.58 . .

C9 C11 O3 114.91 . .

C10 C12 O2 119.58 . .

C10 C12 O4 114.91 . .

O1 C11 O3 125.50 . .

C11 O3 Ag1 134.09 . .

C11 O3 Ag2 105.84 . .

O2 C12 O4 125.50 . .

C12 O4 Ag1 105.84 . .

C12 O4 Ag2 134.09 . .

Ag1 O3 Ag2 101.07 . .

O3 Ag1 O4 78.93 . .

O3 Ag2 O4 78.93 . .

Ag1 O4 Ag2 101.07 . .

loop_

_geom_torsion_atom_site_label_1

_geom_torsion_atom_site_label_2

_geom_torsion_atom_site_label_3

_geom_torsion_atom_site_label_4

_geom_torsion

_geom_torsion_site_symmetry_1

_geom_torsion_site_symmetry_2

_geom_torsion_site_symmetry_3

_geom_torsion_site_symmetry_4

C6 C1 C4 H1 -178.99 1_666 . . .

C6 C1 C4 C9 2.41 1_666 . . .

H6 C1 C4 H1 1.63 . . . .

H6 C1 C4 C9 -176.98 . . . .

C5 C2 C3 H2 -178.99 1_666 . . .

C5 C2 C3 C10 2.41 1_666 . . 2_666

H5 C2 C3 H2 1.63 . . . .

H5 C2 C3 C10 -176.98 . . . 2_666

C2 C3 C10 C7 -3.06 . . 2_666 .

C2 C3 C10 C11 174.60 . . 2_666 .

H2 C3 C10 C7 178.34 . . 2_666 .

H2 C3 C10 C11 -4.01 . . 2_666 .

C1 C4 C9 C8 -3.05 . . . 2_666

C1 C4 C9 C12 174.60 . . . 2_666

H1 C4 C9 C8 178.34 . . . 2_666

H1 C4 C9 C12 -4.01 . . . 2_666

C2 C5 C7 H4 -0.32 1_444 . 2_666 .

C2 C5 C7 C10 -179.31 1_444 . 2_666 .

C6 C5 C7 H4 177.77 2_555 . 2_666 .

C6 C5 C7 C10 -1.23 2_555 . 2_666 .

C1 C6 C8 H3 -0.32 1_444 . . 2_666

C1 C6 C8 C9 -179.31 1_444 . . 2_666

C5 C6 C8 H3 177.77 2_555 . . 2_666

C5 C6 C8 C9 -1.23 2_555 . . 2_666

C5 C7 C10 C3 1.22 2_666 . 2_666 .

C5 C7 C10 C11 -176.54 2_666 . 2_666 .

H4 C7 C10 C3 -179.79 2_666 . 2_666 .

H4 C7 C10 C11 2.46 2_666 . 2_666 .

C6 C8 C9 C12 176.54 . . 2_666 .

H3 C8 C9 C12 -2.46 2_666 . 2_666 .

C4 C9 C8 H3 -179.79 . . 2_666 .

C12 C9 C8 H3 2.46 2_666 . 2_666 .

C4 C9 C12 O1 29.58 . . 2_666 .

C4 C9 C12 O3 -150.96 . . 2_666 .

C8 C9 C12 O1 -152.72 2_666 . 2_666 .

C8 C9 C12 O3 26.74 2_666 . 2_666 .

C4 C10 C7 C5 -1.22 2_666 . 2_666 .

C4 C10 C7 H4 179.79 2_666 . 2_666 .

C11 C10 C7 C5 176.54 2_666 . 2_666 .

C11 C10 C7 H4 -2.46 2_666 . 2_666 .

C4 C10 C11 O2 -29.58 2_666 . 2_666 .

C4 C10 C11 O4 150.96 2_666 . 2_666 .

C7 C10 C11 O2 152.72 2_666 . 2_666 .

C7 C10 C11 O4 -26.74 2_666 . 2_666 .

O2 C11 C10 C3 29.58 2_666 . 2_666 .

O2 C11 C10 C7 -152.72 2_666 . 2_666 .

O4 C11 C10 C3 -150.96 2_666 . 2_666 .

O4 C11 C10 C7 26.74 2_666 . 2_666 .

C10 C11 O4 Ag1 -165.91 2_666 . 2_666 .

C10 C11 O4 Ag2 70.53 2_666 . 2_666 .

O2 C11 O4 Ag1 13.52 2_666 . 2_666 .

O2 C11 O4 Ag2 -110.05 2_666 . 2_666 .

O1 C12 C9 C8 152.72 2_666 . 2_666 .

O3 C12 C9 C8 -26.74 2_666 . 2_666 .

C9 C12 O3 Ag1 -70.53 2_666 . 2_666 .

C9 C12 O3 Ag2 165.91 2_666 . 2_666 .

O1 C12 O3 Ag1 110.05 2_666 . 2_666 .

O1 C12 O3 Ag2 -13.51 2_666 . 2_666 .

Ag1 O1 C12 C9 173.15 2_665 . 2_666 .

Ag1 O1 C12 O3 -6.25 2_665 . 2_666 .

Ag2 O1 C12 C9 -28.05 2_676 . 2_666 .

Ag2 O1 C12 O3 152.56 2_676 . 2_666 .

Ag1 O2 C11 C10 -173.15 1_556 . 2_666 .

Ag1 O2 C11 O4 6.25 1_556 . 2_666 .

Ag2 O2 C11 C10 28.05 1_545 . 2_666 .

Ag2 O2 C11 O4 -152.56 1_545 . 2_666 .

Ag1 O3 C12 C9 70.53 2_666 . 2_666 .

Ag1 O3 C12 O1 -110.05 2_666 . 2_666 .

Ag2 O3 C12 C9 -165.91 2_666 . 2_666 .

Ag2 O3 C12 O1 13.51 2_666 . 2_666 .

C12 O3 Ag1 O4 142.42 2_666 . 2_666 .

Ag2 O3 Ag1 O4 -0.02 2_666 . 2_666 .

C12 O3 Ag2 O4 -125.23 2_666 . 2_666 .

Ag1 O3 Ag2 O4 0.02 2_666 . 2_666 .

Ag1 O4 C11 C10 165.91 2_666 . 2_666 .

Ag1 O4 C11 O2 -13.52 2_666 . 2_666 .

Ag2 O4 C11 C10 -70.53 2_666 . 2_666 .

Ag2 O4 C11 O2 110.04 2_666 . 2_666 .

C11 O4 Ag1 O3 125.23 2_666 . 2_666 .

Ag2 O4 Ag1 O3 0.00 2_666 . 2_666 .

C11 O4 Ag2 O3 -142.42 2_666 . 2_666 .

Ag1 O4 Ag2 O3 0.00 2_666 . 2_666 .

O1 Ag1 O3 C12 -23.11 1_545 . 2_666 .

O1 Ag1 O3 Ag2 119.31 1_545 . 2_666 .

O1 Ag1 O3 C12 54.79 2_665 . 2_666 .

O1 Ag1 O3 Ag2 -162.78 2_665 . 2_666 .

O4 Ag1 O3 C12 -142.42 2_666 . 2_666 .

O4 Ag1 O3 Ag2 0.02 2_666 . 2_666 .

O1 Ag1 O4 C11 156.50 1_545 . 2_666 .

O1 Ag1 O4 Ag2 -78.27 1_545 . 2_666 .

O1 Ag1 O4 C11 -21.99 2_665 . 2_666 .

O1 Ag1 O4 Ag2 103.23 2_665 . 2_666 .

O3 Ag1 O4 C11 -125.23 2_666 . 2_666 .

O3 Ag1 O4 Ag2 0.00 2_666 . 2_666 .

O1 Ag2 O3 C12 -156.50 2_676 . 2_666 .

O1 Ag2 O3 Ag1 78.27 2_676 . 2_666 .

O2 Ag2 O3 C12 21.99 2_667 . 2_666 .

O2 Ag2 O3 Ag1 -103.23 2_667 . 2_666 .

O4 Ag2 O3 C12 125.23 2_666 . 2_666 .

O4 Ag2 O3 Ag1 -0.03 2_666 . 2_666 .

O1 Ag2 O4 C11 23.11 2_676 . 2_666 .

O1 Ag2 O4 Ag1 -119.31 2_676 . 2_666 .

O2 Ag2 O4 C11 -54.79 2_667 . 2_666 .

O2 Ag2 O4 Ag1 162.78 2_667 . 2_666 .

O3 Ag2 O4 C11 142.42 2_666 . 2_666 .

O3 Ag2 O4 Ag1 0.00 2_666 . 2_666 .
